# Enhancing Waveguide Performance in La^3+^-Doped Tellurite Glasses: Energy-Induced Structural Tuning for Reduced Propagation Loss

**DOI:** 10.1021/acsomega.5c02610

**Published:** 2025-05-29

**Authors:** José Luis Clabel Huamán, Kelly Tasso de Paula, Filipe Assis Couto, Gaston Lozano Calderón, José Dirceu Vollet-Filho, Cleber Renato Mendonça

**Affiliations:** São Carlos Institute of Physics, 117186 University of São Paulo, P.O. Box 369, 13560-970 São Carlos, SP, Brazil

## Abstract

Femtosecond (fs) laser irradiation of La^3+^-doped tellurium–zinc (TZL) glass induces structural transformations within the glass surface or volume, resulting in modified chemical compositions and network structures distinct from those of the bulk material. Fs-laser processing promotes the formation of TeO_4_ by transforming TeO_3_ with nonbridging oxygens (NBOs), stabilizing the network and reducing susceptibility to further structural rearrangements. Techniques such as Raman spectroscopy, SEM, and optical microscopy were used to investigate these structural changes and analyze the effects of La^3+^ doping, with a particular focus on identifying TeO_3_ and TeO_4_ bonds and their impact on waveguide optical properties. Conventional methods for characterizing glass surface modifications often lack the sensitivity to capture the extensive, three-dimensional changes induced by femtosecond laser processing, underscoring the need for comprehensive spectroscopic and optical analyses. Using confocal 2D Raman spectroscopy and propagation loss measurements, we examined the laser-modified regions in the TZL glass waveguides. We found that structural changes driven by La^3+^ concentration and the *I*(TeO_3_)/*I*(TeO_4_) ratio significantly influence light confinement and scattering. Complementary simulations validated these trends analytically; modeled electric field and refractive index profiles quantitatively confirmed that energy-induced densification in TeO_4_-rich regions enhances mode confinement and reduces propagation loss. Reduced propagation losses were observed in TeO_4_-rich regions (TZL9), whereas higher losses occurred in TeO_3_-rich regions (TZL5), highlighting the effectiveness of compositional tuning in enhancing waveguide performance through La^3+^-induced structural modifications. This represents a significant advance over previous studies by quantitatively correlating spectroscopic structural changes via the *I*(TeO_3_)/*I*(TeO_4_) ratio with waveguide optical performance. This ability to achieve low-loss waveguides through targeted structural adjustments in tellurite-based glasses offers promising applications in advanced photonic devices, such as all-optical switches and modulators, that require precise control over the optical loss and mode confinement.

## Graphical abstract

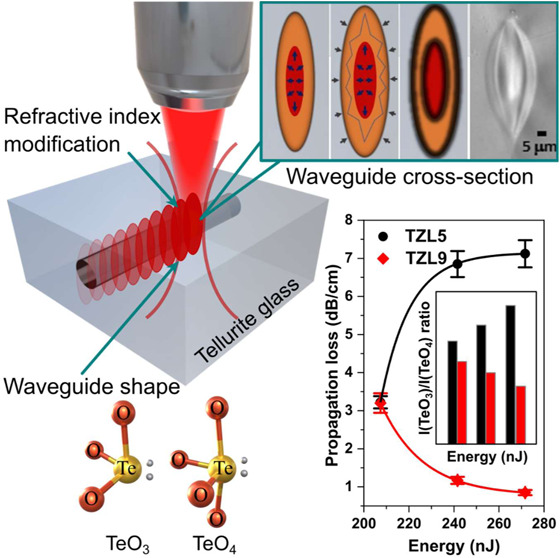

## 1 Introduction

Waveguides are fundamental in modern photonic applications, allowing for the controlled transmission and manipulation of light within a confined structure.^1,2^ These structures are critical in optical communications, sensors, and integrated optics, where precision and efficiency are paramount.^3–5^ The fabrication of high-quality optical waveguides depends not only on the external geometry of the material but also on intrinsic factors such as surface roughness and chemical composition.^6,7^ These factors influence light propagation, optical losses, and the overall performance of the waveguide. In particular, the presence of nonbridging oxygens (NBOs) in the glass matrix can significantly alter the material’s polarizability, enhancing its ability to confine light and reducing scattering losses.^8^

Tellurite–zinc glasses doped with lanthanum (La^3+^) present a high refractive index, broad infrared transparency, and excellent nonlinear optical properties, making them ideal for optical amplifiers and laser systems.^9,10^ La^3+^ further enhances these attributes by modifying the glass network, improving the optical performance. Compared to other glass systems like phosphate or borosilicate, tellurite glasses offer superior infrared transmission and higher nonlinear coefficients, positioning them as promising candidates for advanced photonic devices.^11–13^

In addition, previous studies have shown that La^3+^ enhances network stability and nonlinear optical properties in tellurite glasses through structural reorganization.^14^ However, few reports have systematically explored how La^3+^ content affects the relationship between Raman structural changes and waveguide propagation loss, motivating our current investigation.

The fs-laser micromachining process is key in determining optical losses in waveguides. Parameters such as laser repetition rate and scanning speed are critical in minimizing losses.^15,16^ In terms of repetition rate, two regimes can be identified: (1) In the low repetition rate regime, material changes are primarily induced by individual pulses, which also help minimize heat accumulation, (2) At higher repetition rates, thermal effects become significant because the time interval between pulses is shorter than the thermal diffusion time of silica glass.^16–18^ The investigations in this article will focus on the glass surface under both low and high-repetition rate regimes, while the interior of the glass will be examined under the high-repetition rate regime. Similarly, faster scanning speeds produce smoother surfaces but may require higher pulse energy for proper material modification.^18^ Thus, depending on the exposure parameters, three qualitatively different types of structural changes can be induced in tellurite glasses: (1) an isotropic positive refractive index change, (2) an induced birefringence with a negative index change, and (3) voids. A balance between the laser irradiation parameters is essential for optimizing the waveguide performance and minimizing scattering losses.

Lanthanum-doped tellurite–zinc glasses have distinct advantages in waveguide fabrication due to the structural modifications introduced by La^3+^, which improve the glass’s response to laser-induced changes.^9^ These modifications also enhance the material’s thermal and mechanical stability, which is essential for applications such as telecommunications, optical amplifiers, and integrated photonic circuits. While the exact relationship between glass composition and optical losses warrants further investigation, this work explores the influence of La^3+^ concentration on waveguide performance.^10^

This work focuses on tellurite–zinc glasses, doped with lanthanum (La^3+^), which exhibit a strong nonlinear optical response. Using femtosecond laser pulses at 1030 nm, we successfully fabricated surface line patterns and waveguides with micrometer-scale resolution. Our findings demonstrate that different concentrations of La^3+^ ions influence the long-range quality of the patterns, both on the surface and within the volume of the TZL glasses. Raman confocal spectroscopy reveals that the micropatterning process significantly reduces the *I*(TeO_3_)/*I*(TeO_4_) ratio, in both the surface and volume, as compared to that of nonpatterned. Furthermore, optical loss measurements indicate that waveguide performance is closely related to the La^3+^ ion concentration and the change in the *I*(TeO_3_)/*I*(TeO_4_) ratio. These results highlight an efficient and cost-effective approach for fabricating TZL glass patterns, potentially expanding its use in photonic devices.

## 2 Experimental Section

### 2.1 Preparation of the TZL Glass

The melt-quenching technique was used to fabricate TeO_2(0.7)_–ZnO_(0.3–*x*)_–La_3_O_2(*x*)_ glasses with *x* = 0.05, 0.07, and 0.09 (*x* in mol %); these samples are referred to as TZL5, TZL7, and TZL9, respectively, adding all chemical powders according to the designed proportion of the glass composition. Subsequently, the mixture was transferred to a platinum crucible in an electric furnace at 850 °C for 45 min and periodically stirred to ensure homogenization. Afterward, the samples were molten with thermal treatment at 340 °C for 3 h and then slowly cooled to room temperature. All samples were cut into circular shapes (1.5 cm diameter) and polished until about 1.0 mm thick. The preparation procedure followed previous work on tellurite systems.^14,19^

### 2.2 Characterization

The morphology of the fabricated microstructures was characterized by scanning electron microscopy (SEM, FEI Inspect-F50) and atomic force microscopy (AFM, Nanosurf easyScan 2). The initial glass surface roughness was determined by AFM, yielding a root-mean-square (RMS) roughness of 2.3 nm. A confocal Raman microscope (Witec-Alpha-300R) with a diode laser at a wavelength of 532 nm was focused on the sample using a 100× objective to measure micro-Raman spectra in backscattering geometry and to identify the phase transformation.

### 2.3 Femtosecond Laser Micromachining

The fs-laser micromachining was realized using a diode-pumped Yb:KGW laser system that delivers 216 fs pulses centered at 1030 nm, focused on the TZL glasses by a 0.65 numerical aperture microscope objective. The microfabrication was studied by varying the repetition rate from 20 Hz to 200 kHz, while adjusting the scanning speeds to 12.5 and 25 μm/s, facilitated by precise x–y–z translation stages that positioned the samples. A CCD camera coupled with backlight illumination enabled real-time monitoring of the micromachining process. All experiments were conducted in ambient air at room temperature under standard atmospheric conditions.

Laser micromachining was conducted with varying pulse energies (*E*_0_) and a range of pulses per spot (*N*), from 1 up to approximately 10,000. The number of pulses (*N*) was controlled by adjusting the laser’s repetition rate and the scanning speed of the translation stage. For each value of *N*, sets of 400 μm-long lines, spaced 20 μm apart, were fabricated to assess the impact of the pulse energy on line width.

Laser micromachining for Type I waveguide fabrication was performed by focusing the laser beam into the glass volume using a microscope objective with various pulse energies applied to achieve the desired modification.

We measured the waveguide near-field mode intensity using a standard end-face coupling setup. An objective lens (40×, NA = 0,65) was employed to couple the 633 nm laser beam into the input port of the fabricated waveguide. An imaging system composed of an objective lens (20×, NA = 0.40) and a CCD camera recorded the near-field mode intensity distribution at the output port of the waveguide. The setup mentioned above was mounted in motion stages for precise adjustment. The simulations were performed using the finite difference time domain (FDTD) solver, Tidy3D, which numerically solves Maxwell’s equations to model electromagnetic wave behavior. The model represents a waveguide cross section, where the software calculates the electric field distribution and the effective refractive index of the guided modes.

## 3 Results and Discussion

### 3.1 Scanning Electron Microscopy (SEM) and Energy-Dispersive X-ray (EDX) Spectroscopy

Figure 1 shows SEM and EDS mappings of four glass components (Te, Zn, and La atoms) of the micropatterned structure on TZL5, which are representative of the consistent behavior observed across all three studied glasses. The SEM images of the TZL5 glass microstructures applied to 25 pulses and different pulse energies are shown in Figure 1a. From the figure, the surfaces are smooth except in the microfabricated region. On the other hand, no second phases, bubbles, or clusters were observed, which is important for optical applications that cannot bear large scattering losses, which are observed from SEM analysis. The topography of the microfabricated pattern shows that these grooves are narrow, down to 2.5 μm. Besides, the microfabricated pattern appears very uniform over longer distances. The interaction of the laser and glass surface in localized areas is driven by the intense laser irradiation, as revealed by the effect of the pulse energy (from 100 to 305 nJ) on the line width of micromachined grooves by fs-laser for TZL5 glass. When the pulse energy is increased from 100 nJ to 305 nJ, the line width increases from 1.1 to 2.5 μm. However, as the grooves increase, their edges become rougher, compromising the overall quality. This suggests that the patterned lines, fabricated with energies lower than 250 nJ, are of high quality, without any notable residue between the lines, even for the imprinted patterns. This indicates that it is essential to fabricate the photonic devices using proper laser parameters to avoid harmful cracks.

**Figure 1:**
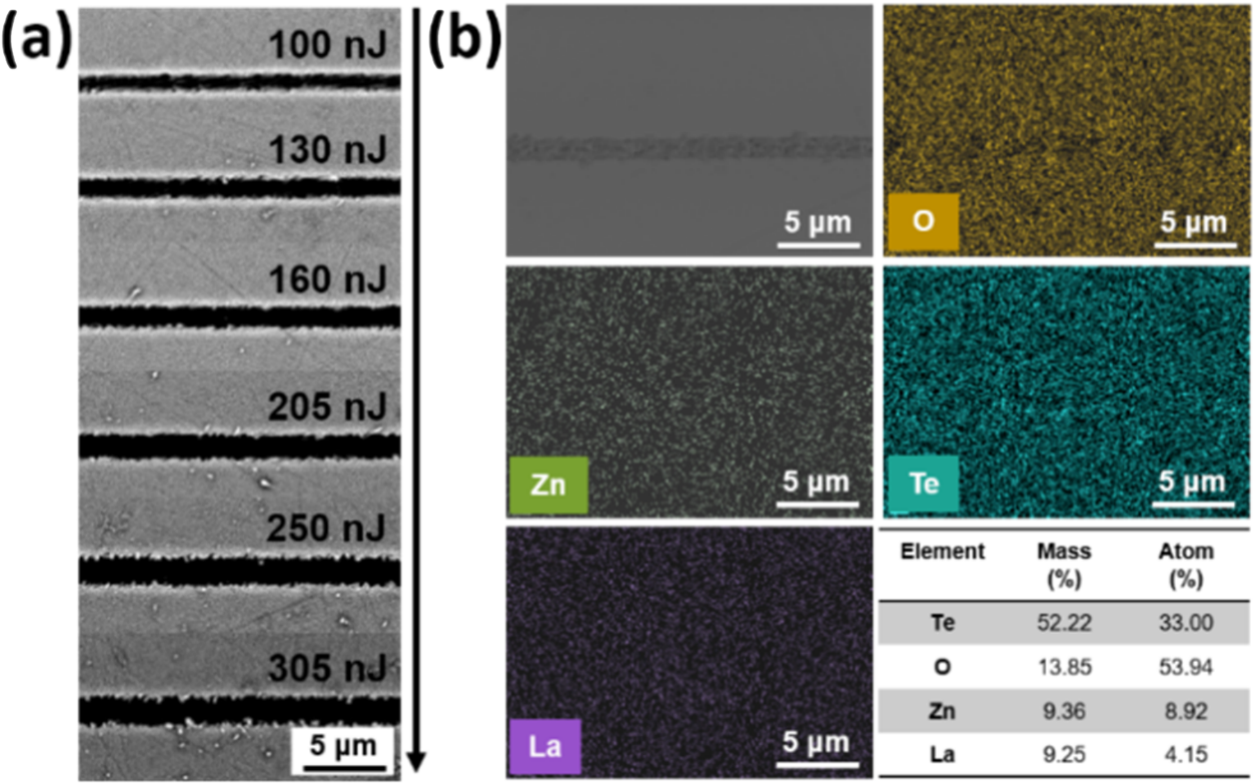
(a) SEM image of the microfabricated lines on TZL5 glass using 25 pulses, varying laser pulse energies from 100 to 305 nJ. (b) Elemental mapping (O, Zn, Te, La) of the microfabricated regions, along with the corresponding element composition table showing mass and atomic percentages.

The elemental analysis of La^3+^-doped TZL5 glass suggests not only the existence of Te, Zn, and O but also the signal of the La element is acquired, as shown in Figure 2b. The corresponding images of the elemental mapping confirmed that the TZL5 glass was uniformly distributed over the surface, indicating an efficient insertion of La^3+^ ions into the TZL5 glass. The EDS analysis detected no other elements or impurities. However, a deficiency of oxygen was observed in microfabricated regions. This oxygen deficiency contributed to the break of the bond of the TZL5 glass structure, leading to structural transformation units, as demonstrated in the Raman results (presented later).

**Figure 2:**
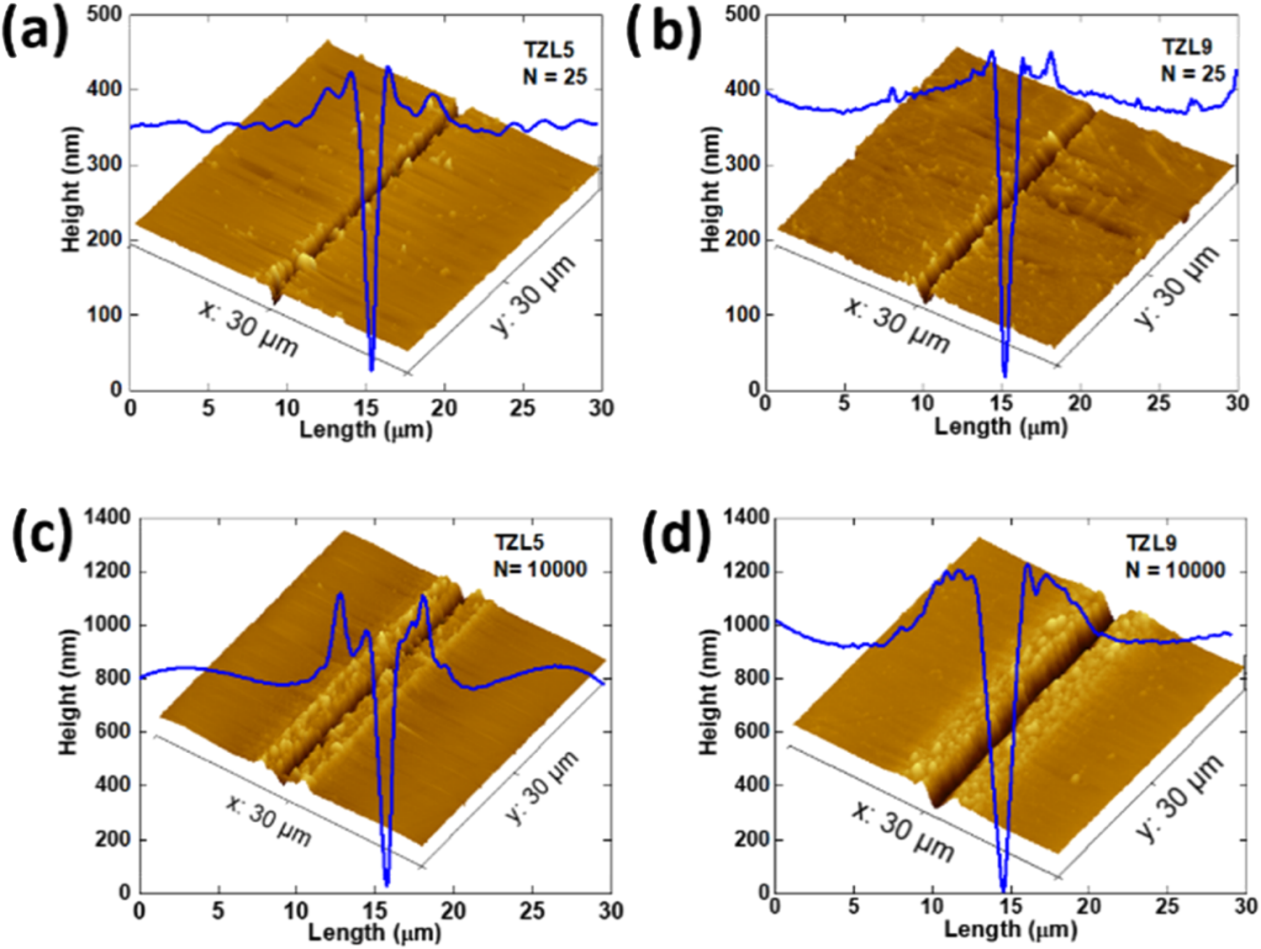
Atomic force microscopy (AFM) 3D surface profiles of microfabricated regions in (a) TZL5 at *N* = 25, (b) TZL9 at *N* = 25, (c) TZL5 at *N* = 10,000, and (d) TZL9 at *N* = 10,000. The horizontal topography profiles through the cavity center (blue line) are shown above each 3D surface, highlighting the differences in roughness and surface morphology between the two glasses.

### 3.2 Atomic Force Microscopy (AFM)

AFM measurements were used to verify the surface cavities, surface roughness (*R*_a_), and morphology of the TZL glasses with different La_2_O_3_ concentrations. Figure 2 shows a clear contrast between the microfabricated lines and the background, with image (a) showing TZL5 and image (b) showing TZL9, both fabricated using 25 pulses. No significant height variation was observed along the channel length for any structures at the pulse numbers used. The surface roughness measured outside the grooves is 14 nm for TZL5, 13 nm for TZL7, and 13.4 nm for TZL9, indicating similar values across samples. However, these values are higher than the 1.78 nm recorded for tellurium–zinc glass and perovskite particles embedded in tellurium–zinc glass.^20^ This increased roughness in TZL glasses is likely due to intrinsic structural differences, possibly related to the La_2_O_3_ content, phase separation, or localized crystallization during the cooling process, resulting in a less uniform surface. However, lower surface roughness in TZL glasses could reduce scattering and enhance light propagation in microfabricated waveguides, as discussed in later sections.

The AFM profiles of TZL glasses reveal a clear relationship between the number of pulses and the ablation depth, determined at a laser energy of 305 nJ for both low (*N* = 25) and high (*N* = 10,000) pulse numbers. At *N* = 25, TZL5 and TZL9 exhibit depths of 350 and 380 nm, respectively, with a characteristic V-shaped cross-sectional profile that persists at both low and high pulse numbers and a gradual reduction in depth toward the cavity edges. However, subtle irregularities appear at the groove edges, even at low pulses.

As the pulse number increases to *N* = 10,000, the grooves deepen to 900 nm in TZL5 and 1050 nm in TZL9, with a marked increase in roughness, particularly in TZL5. The more pronounced irregular edge step in TZL9 at higher pulse numbers suggests a “balling effect,” where molten material from the laser ablation process resolidifies unevenly, forming edge irregularities.^20^ This effect likely results from thermal instability during ablation, leading to uneven material redistribution along the cavity boundaries.^21^ The differing responses of the two glasses at high pulse counts highlight how laser exposure intensifies surface roughness and edge irregularities, with TZL9 showing more significant changes at higher pulse numbers.

### 3.3 Raman Spectra of TZL Glasses

Figure 3 shows Raman spectra at room temperature and their deconvolution. The Raman modes related to the bands allow describing the structural behavior of the glass network in relation to the La_2_O_3_ concentration. The bands between 550 and 1100 cm^–1^, fitted with Gaussian functions, are attributed to tellurite structural units: TeO_4_ (625 and 677 cm^–1^), TeO_3_ (789 cm^–1^), and TeO_3+1_ (747 cm^–1^), with the presence of nonbridging oxygen (NBO) in the TeO_3_ and TeO_3+1_ units.^14^ Additionally, La^3+^ ions act as network modifiers in the glass matrix, due to their larger ionic radius, disrupting Zn–O coordination and leading to a diverse distribution of TeO_*x*_ polyhedra.^22^ Consequently, bands around 378 and 469 cm^–1^ correspond to Te–O–La linkages, bending vibrations involving NBOs in TeO_3_, and Te–O–Te bending vibrations [REF]. This pattern aligns with reports for other tellurite-based glasses, where modifiers like La_2_O_3_ disrupt the Te–O–Te network and enhance the presence of NBOs, as seen in borotellurite and boro-phosphate systems [REF].

**Figure 3:**
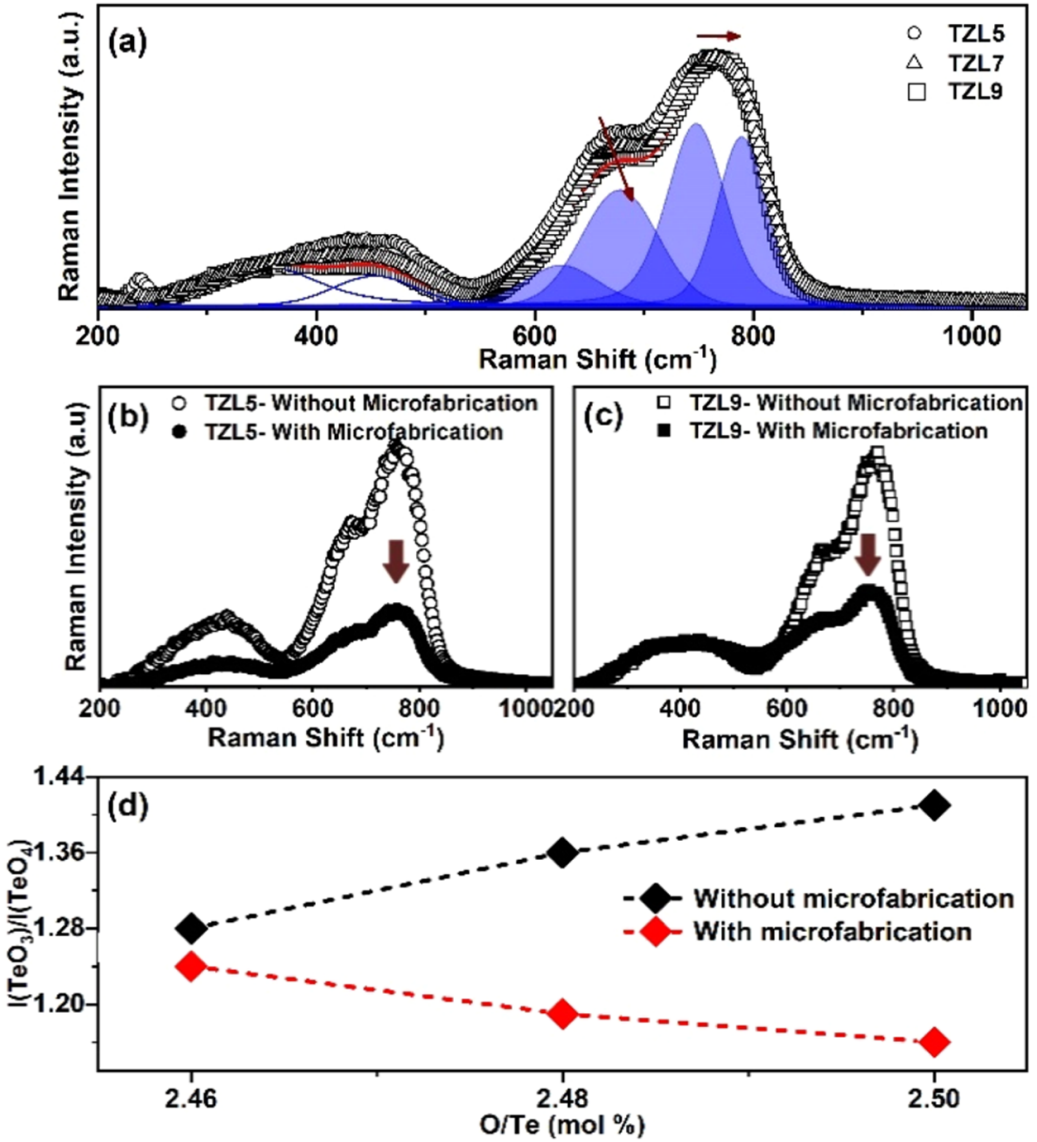
Raman spectra of TZL glasses. (a) Deconvoluted Raman spectra of TZL5, TZL7, and TZL9 glasses, highlighting the different vibrational modes. (b) Raman spectra of TZL5 in nonmicrofabricated (open circles) and microfabricated regions (filled circles). (c) Raman spectra of TZL9 in nonmicrofabricated (open squares) and microfabricated regions (filled squares). (d) Ratio of *I*(TeO_3_)/*I*(TeO_4_) as a function of O/Te (mol %) for both microfabricated and nonmicrofabricated regions.

The peak at ∼378 cm^–1^ shifts linearly toward lower wavenumber with increasing La_2_O_3_ concentration, likely due to La–O vibrations within the network. Studies indicate that rare-earth oxides like La_2_O_3_ exhibit Raman bands below 420 cm^–1^, with bands around 360 cm^–1^ attributed to La–O and LaO_n_ polyhedral vibrations.^22^ The shift to lower wavenumbers reflects the incorporation of La^3+^, which elongates the Te–O bonds and relaxes the TeO_4_ tetrahedra. According to Sakka et al.,^23^ there is an inverse relationship between Te–O bond length and peak wavenumber: peaks shift to lower wavenumbers as the shortest Te–O distance increases. Additionally, the bands between 550 and 1100 cm^–1^ shift to higher wavenumbers with La doping, indicating instability in the long Te–O bonds, as shown in Figure 3a.

Figure 3b,c compares the Raman spectra of TZL5 and TZL9 in regions with and without microfabrication on the surface glass. Shifts in Raman peaks reflect structural changes induced by irradiation, along with variations in peak intensity. In the microfabricated region, the 378 cm^–1^ peak shifts by 11.2 cm^–1^, while the TeO_4_ and TeO_3_ peaks shift by 8.3 and 9.2 cm^–1^, respectively, indicating structural rearrangements. The reduction in the TeO_3_ and TeO_4_ intensity after femtosecond laser microfabrication suggests that laser ablation breaks weaker bonds, converting or eliminating TeO_3_ units.

To further analyze these transformations, the intensity ratio *I*(TeO_3_)/*I*(TeO_4_) was calculated to assess short-range structural changes as La_2_O_3_ concentration increases, reflecting the formation of nonbridging oxygens (NBOs). For surfaces without microfabrication, Figure 3d shows this trend, with *I*(TeO_3_)/*I*(TeO_4_) ratio increasing from 5 to 9 mol % La_2_O_3_, indicating network reorganization and enhanced polarizability.^14^ The increase in the *I*(TeO_3_)/*I*(TeO_4_) ratio for O/Te (mol %) at 2.50, comparing the samples before and after microfabrication, was approximately 21%. This trend aligns with observations by Abul-Magd et al.,^24^ who reported similar structural transitions in La_2_O_3_-doped borate glasses, where La^3+^ ions created more NBOs, disrupting the network structure. An inverse behavior is observed for the surface with microfabrication, indicating a reorganization of the tellurite network. This leads to a decrease in the *I*(TeO_3_)/*I*(TeO_4_) ratio by promoting the formation of TeO_4_ over TeO_3_. Although relaxation and thermal stress may cause minor changes, these factors are less likely to drive TeO_3_ to TeO_4_ conversion due to minimal heat accumulation in ultrashort laser pulses. However, this process can be induced by a combination of thermal effects and high-pressure shock waves that can promote such structural formation of TeO_4_ to TeO_3_.

Studies by Khanna et al.^25^ showed that under high pressure (>1.19 GPa) in tellurite glass, a similar structural transformation can occur: TeO_3_ + NBO → TeO_4_. Lee et al.^26^ observed analogous pressure-induced changes in borate glasses (BO_3_ + NBO → BO_4_), and ab initio molecular dynamics simulations have confirmed these results in borate, silica, and aluminosilicate systems.^27^ Femtosecond laser microfabrication, which induces minimal thermal effects, can generate high-pressure shock waves even at low pulse energies due to the ultrashort pulse width.^28^ Shockwave pressures have been estimated up to 10 GPa for energies between 4.5 and 70 nJ within nanoseconds, pulses, reaching 172.3 GPa over 30 ps.^28,29^ Direct fs-laser irradiation can yield pressures from 100 to 300 GPa,^30^ similar to the extreme conditions in materials like stishovite, a high-pressure form of silica, which undergoes lattice-level transformations at pressures above 300 GPa.^31^ This highlights the critical role of high-pressure behavior in understanding structural changes in materials including glasses.

Thus, the predominant mechanisms are structural transformation and NBO formation, reducing the *I*(TeO_3_)/*I*(TeO_4_) ratio and reflecting the material’s response to laser-induced modifications. The laser-induced conversion of TeO_3_ into TeO_4_ impacts optical waveguide fabrication and may influence optical coupling losses. This relationship between the glass structure and waveguide performance will be further analyzed in subsequent sections.

### 3.4 Femtosecond Laser Micromachining and Incubation Processing

Figure 4a,c displays optical microscopy images of microfabricated lines on TZL5 and TZL9 glasses, produced using 25 pulses and laser pulse energies ranging from 100 to 305 nJ. Figure 4b,d further plots the line radius squared (*r* ^2^) against pulse energy (*E*_0_) in log scale, illustrating how ablation width expands with increasing energy. To estimate the threshold energy (*E*_th_) and Gaussian beam radius (*w*_0_), we model the relationship between *r* ^2^ and *E* using

1 $$r^{2}=\frac{w_{0}^{2}}{2}\ln\left(\frac{E_{0}}{E_{\mathrm{th}}}\right)$$

Fitting this model to these specific data sets yielded the following values: for TZL5, *E*_th_ = 69 nJ and *w*_0_ = 1.2 μm, while for TZL9, *E*_th_ = 74 nJ and *w*_0_ = 1.2 μm. The slightly higher *E*_th_ for TZL9 suggests that more energy is needed to initiate ablation, likely because its lower *I*(TeO_3_)/*I*(TeO_4_) ratio makes the glass network more flexible. This flexibility resists ablation, requiring more energy than TZL5, which has a higher *I*(TeO_3_)/*I*(TeO_4_) ratio, creating a more rigid network that ablates more easily.

**Figure 4:**
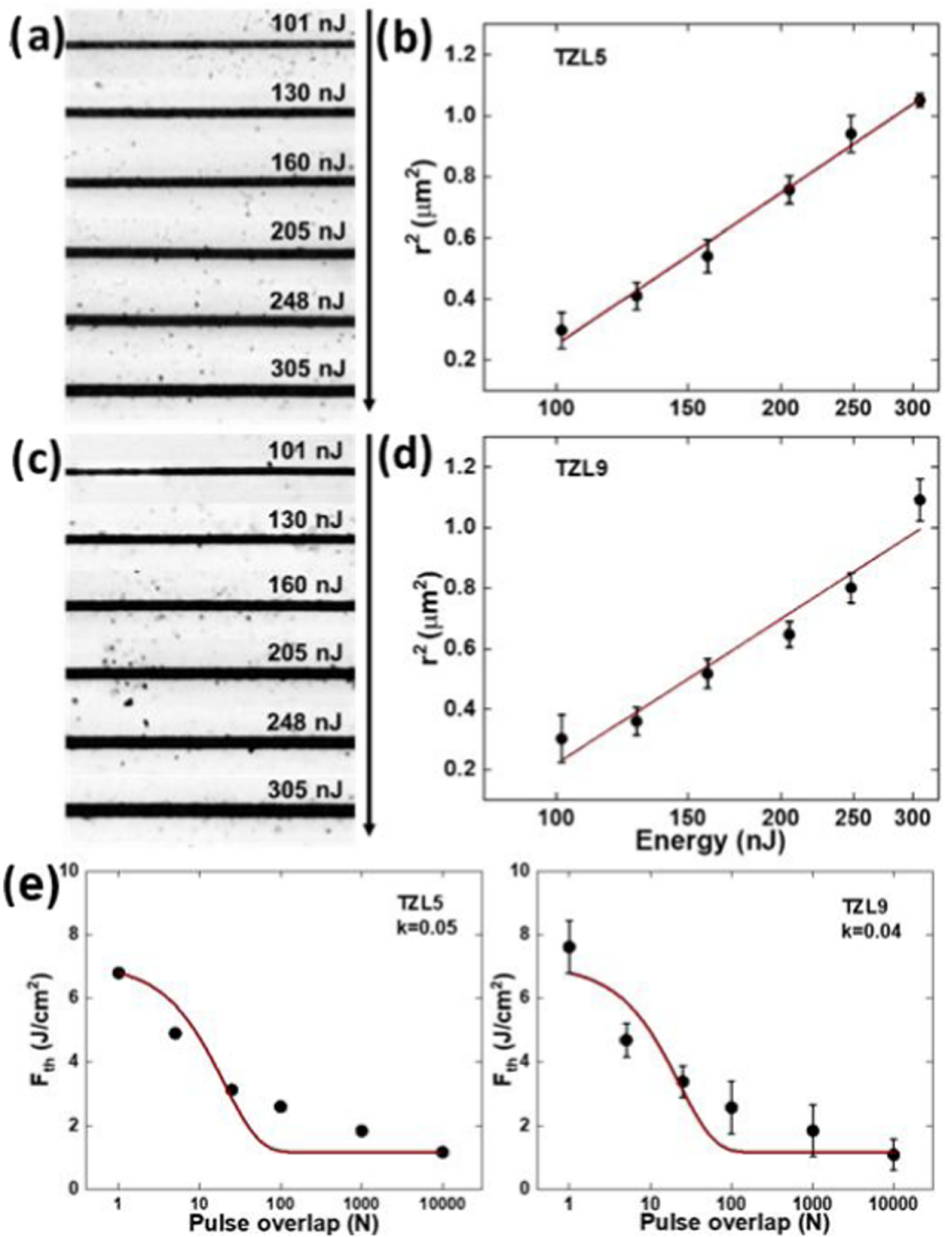
For 25 pulses (*N* = 25). (a) Optical microscopy images of TZL5 micromachined lines at different pulse energies. (b) The respective line radius squares as a function of the pulse energy. (c) Optical microscopy images of TZL9 micromachined lines at different pulse energies. (d) The respective line radius squares as a function of the pulse energy. (e) Incubation curves for TZL5 and TZL9.

The Gaussian beam radius for both TZL glasses shows only a slight variation, suggesting a mostly uniform energy distribution from the laser. Despite the structural differences between samples, the nearly uniform *w*_0_ shows similar laser-material interaction dynamics. In a broader range of experiments, with the number of pulses varying from 10,000 to 1, the average beam radius ranged from approximately 0.9 to 1.2 μm. Threshold energies were determined for each pulse variation, ranging from 16 to 106 nJ for TZL5 and 14 to 129 nJ for TZL9, as the pulse count decreased from 10,000 to 1. As expected, the *r* ^2^ versus pulse energy plots exhibit a logarithmic increase in the ablation width with increasing energy.

Our findings indicate that the threshold energy necessary for damage decreases with an increasing number of laser pulses due to the cumulative heat accumulation effect, which reduces the need for high energy per pulse as more pulses are applied. This incubation effect, observed in various materials such as semiconductors,^32^ polymers,^33^ ceramics,^34^ and glasses,^20^ is essential for optimizing laser processing across diverse applications. Although various models explain the incubation effect, the probabilistic defect accumulation model fails to account for the threshold fluence saturation shown in Figure 4e. Thus, the exponential defect accumulation model better interprets our experimental results.

The relationship can be described as follows: the threshold fluence after *N* pulses, *F*_th,*N*_, is related to the single pulse threshold laser fluence, *F*_th,1_, and the infinite pulses threshold fluence, *F*_th,∞,_ using *F*_th,*N*_ = (*F*_th,1_ – *F*_th,∞_)*e* ^–*k*(*N* – 1)^ + *F*_th,∞_, where *k* is the incubation parameter.

Figure 4e presents the incubation curves resulting from the fs-laser micromachining of TZL5 and TZL9. For TZL5, the threshold laser fluence after one pulse (*F*_th,1_) is 68 J/cm^2^, while for TZL9 it is 7.6 J/cm^2^. As the number of pulses increases, both glasses show a decrease in threshold laser fluence, reaching *F*_th,∞_ values of 1.0 J/cm^2^ for TZL5 and 1.1 J/cm^2^ for TZL9. TZL9 slightly higher *F*_th_ suggests a more flexible network, likely due to its lower *I*(TeO_3_)/*I*(TeO_4_) ratio, resulting in a more gradual response to laser pulses. Although the difference is small, it suggests a similar overall ablation behavior for both glasses.

Fitting the data in Figure 4e yielded incubation parameters of 0.05 ± 0.01 for TZL5 and 0.04 ± 0.01 for TZL9, indicating that both materials require a large number of pulses to induce significant damage, reflecting a low incubation parameter. This supports the exponential defect accumulation model, where laser-induced defects increase the material’s susceptibility to further pulses.

The Keldysh parameter (γ) is essential for understanding laser-induced breakdown in transparent materials, offering insights into ionization processes triggered by high-intensity pulses. It differentiates between multiphoton and tunneling ionization by comparing laser intensity to material properties like band gap energy. Tunneling ionization dominates at strong fields and low frequencies, while multiphoton ionization prevails at higher frequencies. The Keldysh parameter is defined by^35^

2 $$\gamma_{K}=(\omega /e)\sqrt{\left(m_{e}\varepsilon_{0}cn_{0}E_{g}\right)/I_{0}}$$

where ω is the laser frequency, *I*_0_ represents the laser intensity at focus, *m*_*e*_ and *e* are the electron’s reduced mass and charge, respectively, *c* is the speed of light, *n*_0_ is the refractive index of the material, *E*_g_ is the band gap energy, and ε_0_ is the vacuum permittivity.

As laser intensity changes, the dominant ionization mechanism shifts: at lower intensities (γ > 1), multiphoton ionization predominates, while at higher intensities (γ < 1), tunneling ionization takes over. This distinction is essential, as each regime produces specific effects such as surface modification or deeper ablation that directly impact microfabrication outcomes. For TZL5 glass, *E*_g_ is 3.53 eV and *n*_0_ is 1.98, while for TZL9 glass, *E*_g_ is 3.61 eV and *n*_0_ is 1.96.^14^ Calculations based on these values and the threshold energies give Keldysh parameters of 0.15 (single pulse) and 0.39 (10,000 pulses) for TZL5 and 0.16 (single pulse) and 0.43 (10,000 pulses) for TZL9. These values indicate tunneling ionization as the dominant mechanism in both single and multiple pulse regimes, typical at high laser intensities, where the electric field enables electrons to tunnel through the Coulomb barrier, defining the material response to the laser.

### 3.5 Optical Waveguides and Implications for Fabrication

Although TZL7 was structurally characterized, propagation losses were not measured for this composition. We selected TZL5 and TZL9 to highlight the contrast between low and high La_2_O_3_ content. Fs-laser-written waveguides are typically classified as Type I, formed by a positive refractive index change in the focal volume, or Type II, created between laser-induced damage tracks due to stress effects.^36^ Figure 5a illustrates the optical setup for inscribing continuous waveguides in TZL glass using an ultrafast femtosecond laser beam focused precisely within the material. Figure 5b shows waveguide formation through multiple laser pulses. Differences in the morphology of microfabricated waveguides are shown in Figure 5a (for TZL5) and Figure 5b (for TZL9), with longitudinal profiles on the left and cross-sectional views on the right. The waveguide core in TZL5 is notably smaller and more constricted than the broader core observed in TZL9.

**Figure 5:**
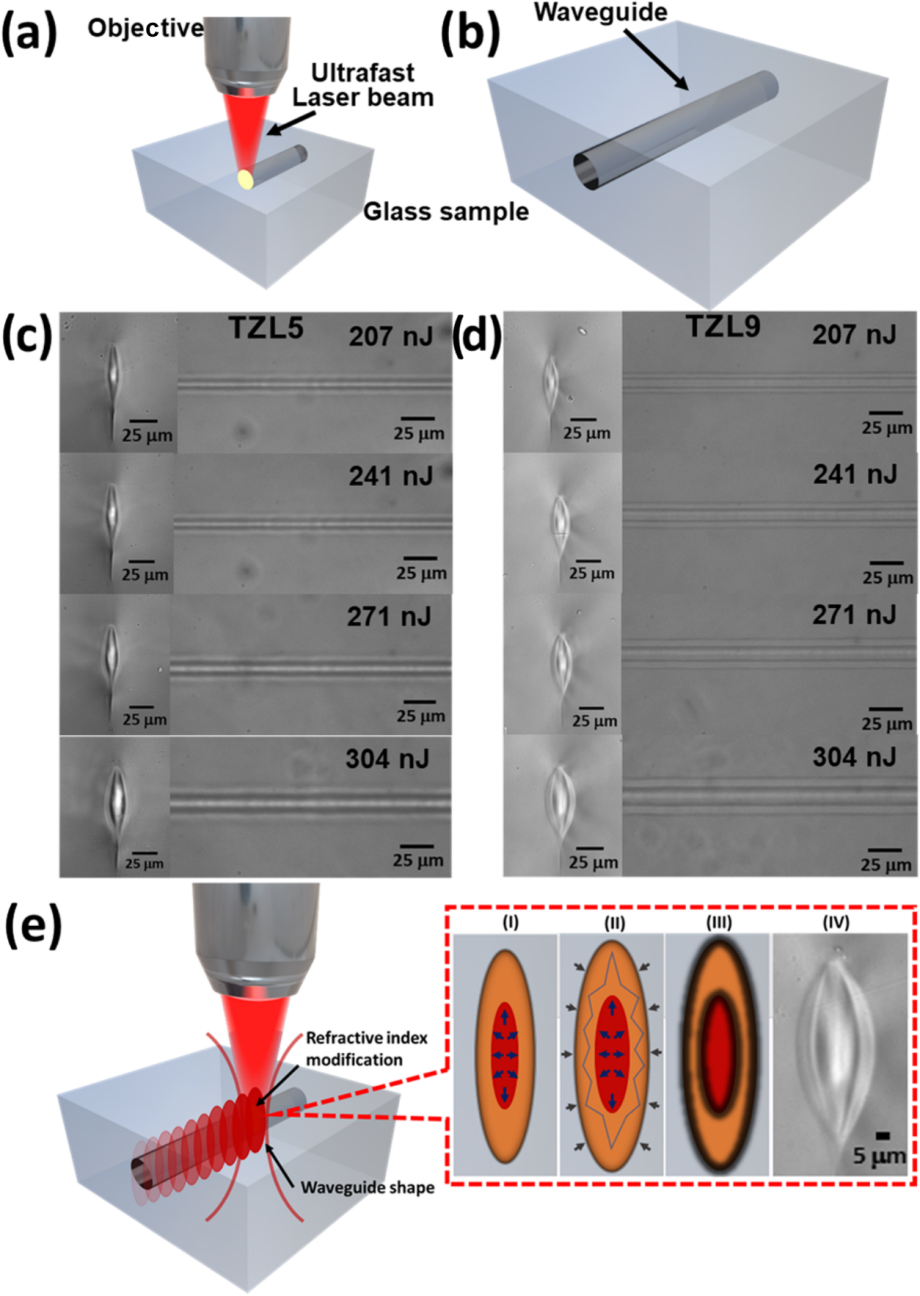
(a) Scheme of the experimental setup for waveguide writing. (b) Formation of a waveguide by multiple pulses. SEM images of microfabricated optical waveguides in (c) TZL5 and (d) TZL9 glasses. The left panels show the cross-sectional views of the waveguides, while the right panels display the longitudinal profiles. (e) Schematic representation of waveguide formation in TZL glass induced by femtosecond laser irradiation. Insets show structural densification and the resulting elliptical cross-sectional morphology.

Waveguide morphology is influenced by parameters such as scan speed, pulse energy, pulse duration, repetition rate, surface roughness, and polarization state.^2,37^ The observed waveguide profiles, narrower in TZL5 and broader in TZL9 (Figure 5c,d), can be correlated with the effect of the *I*(TeO_3_)/*I*(TeO_4_) ratio on structural flexibility previously discussed. Unlike surface microfabrication, where changes are more localized, waveguide formation within the bulk glass involves additional complexity due to shockwave propagation and deeper pressure effects. Thus, waveguide morphology depends strongly on fs-laser processing parameters and the inherent properties of TZL glass. To understand these differences, it is essential to consider how internal laser-induced pressures and the glass network’s response contribute to the final waveguide structure.

Within the volume, femtosecond laser pulses generate significant shock pressures, estimated to reach several tens of GPa,^29,31,38^ leading to structural transformation and NBO formation. In TZL9, with a higher initial *I*(TeO_3_)/*I*(TeO_4_) ratio, these pressures promote the gradual conversion of TeO_3_ into more stable TeO_4_ units. The remaining TeO_3_ units and associated NBOs provide flexibility, allowing for profile expansion under laser-induced pressure and improving the light-guiding efficiency. In contrast, TZL5, with a lower *I*(TeO_3_)/*I*(TeO_4_) ratio, has a more rigid network, with fewer TeO_3_ units available for conversion. While laser-induced shock waves still cause structural changes, the inherent stiffness of TZL5 limits plastic deformation, resulting in a more confined core morphology and reduced flexibility during waveguide formation.^39^ Additionally, the plastic deformation induced by these shock waves within both glasses may leave unique traces, such as dislocation structures, similar to those observed in La-rich (∼10 mol % La_2_O_3_) and La-less (∼0.4 mol % La_2_O_3_) phosphate glass.^40^ Examining the origins of this structural flexibility and core morphology requires considering how local temperature gradients and heat accumulation influence waveguide formation under high-repetition femtosecond laser pulses.^41^

A local temperature gradient mechanism has been proposed for waveguide formation, occurring specifically within the focal region. The microstructural and structural changes induced by the high concentration of laser energy along the waveguide boundaries regulate the temperature gradient, minimizing optical distortions and ensuring efficient energy distribution. This process enables the inscription of waveguides with low optical losses, as observed in the distinct zones within TZL5 and TZL9. The fs-laser inscription was realized at a high-repetition rate regime. At high-repetition rates (>100 kHz), local melting and rapid solidification/quenching lead to increased density and refractive index in the waveguide boundary and core, forming a cross-sectional area larger than the focal spot, as shown in Figure 5e. While local compression may occur, it is not the dominant mechanism, as heat dissipates from the focal volume before the next pulse arrives.^42^ Heat accumulation and diffusion generate pressure waves during waveguide fabrication, causing structural modifications to propagate outward from the hot, pressurized center. This results in a densified cross section with two distinct zones, as seen in Figure 5e, where bond breaking and ion diffusion, including O^2–^, alter the local glass composition.

To support the microstructural analysis of the cross section, we used confocal Raman microscopy to examine the local structural rearrangements in the transverse sections of the waveguide. The Raman spectra obtained in the range from 700 to 910 cm^–1^, corresponding to the TeO_3_ structure, reveal a shift associated with the structural transformation (TeO_3_ + NBO→TeO_4_) induced by fs-laser irradiation, as discussed in the previous section. Figure 6a,b displays the 2D Raman images for TZL5 and TZL9 at 271.6 nJ, respectively, highlighting the TeO_3_ structure along the waveguide cross section. The color contrast reflects specific Raman shifts within the range of 758–765 cm^–1^ for TZL5 and 760–770 cm^–1^ for TZL9, associated with structural transformations in the glass network, Te–O–Te bond breaking, or chemical migration. This shift indicates alterations in bond lengths and densification of the network structure, similar to observations by Fernandez et al.,^40^ in phosphate glass, where Raman shifts were correlated with changes in the average P–O bond length and network contraction. The shift observed here suggests a comparable densification process within the framework influenced by the *I*(TeO_3_)/*I*(TeO_4_) ratio.

**Figure 6:**
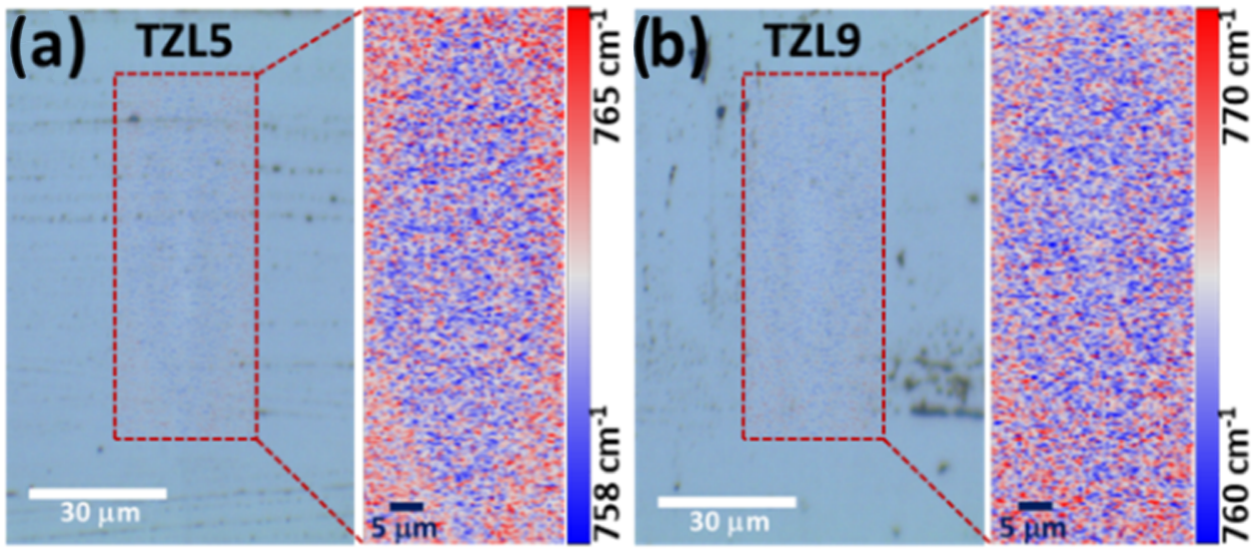
Overlay of optical and confocal Raman microscopy images for TZL5 (a) and TZL9 (b). The magnified 2D Raman mapping for each TZL glass shows the Raman shift in the range from 700 to 910 cm^–1^ after fs-laser irradiation.

Figure 7a,e shows the waveguide profiles for TZL5 and TZL9 based on the optical image. The insets in Figure 7a,e highlight the core and tail regions of the eye-shaped waveguide structures, as revealed in the Raman image. The Raman signal intensity displays an elongated, symmetrical cross-sectional profile with a radial gradient except for a depression at the focal center. This suggests that the irradiated region center may have developed a porous structure with reduced density.^43^ A similar symmetric elongated cross section was recently observed in crystalline tracks written in lead germanate glass.^44^ The overlay of optical and Raman images highlights stress-induced structural changes from the fs-laser, with TZL5 showing a narrower profile and localized structural changes, while TZL9 reveals a broader waveguide and more extensive modification.

**Figure 7:**
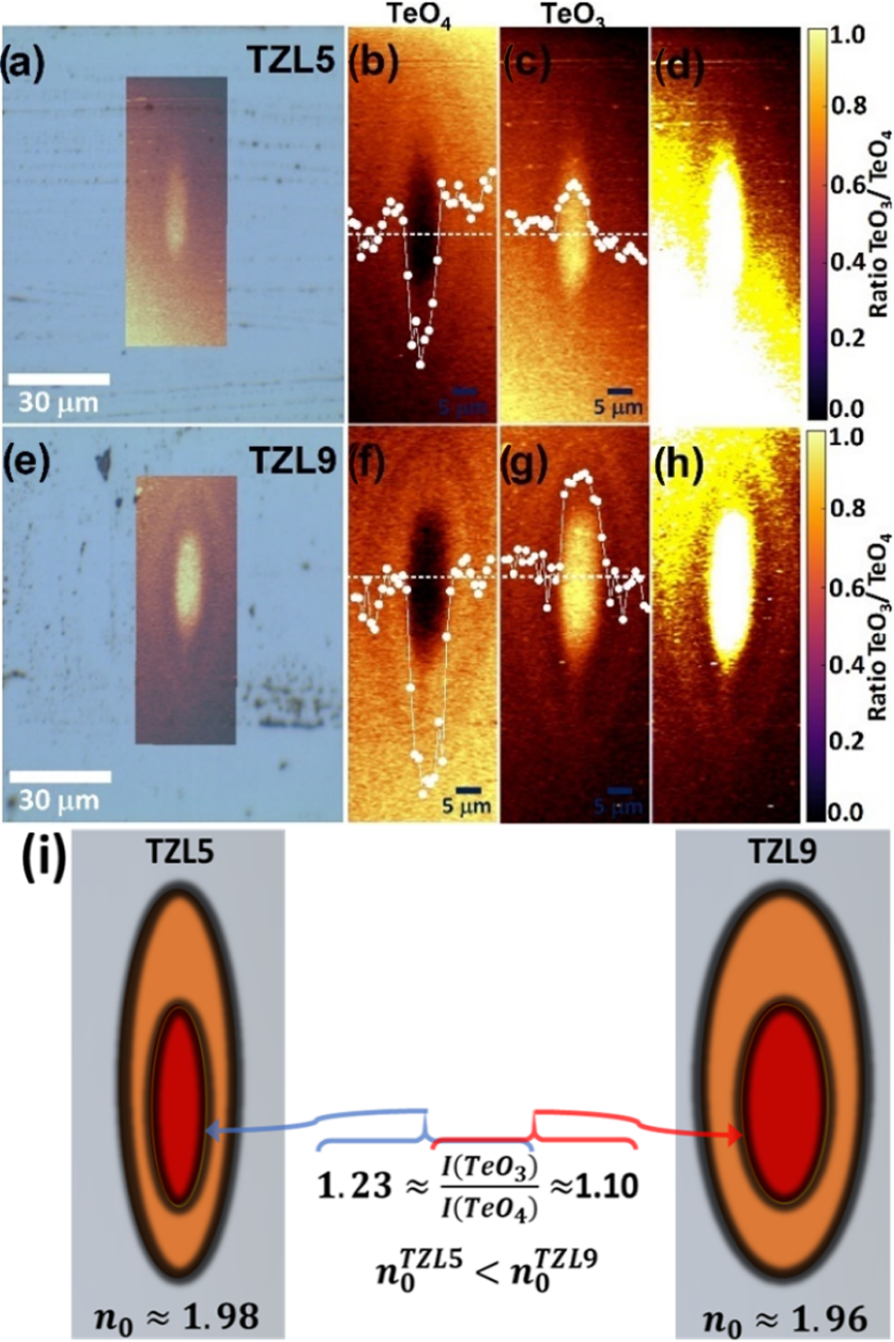
Overlay of optical and confocal Raman microscopy images for TZL5 (a) and TZL9 (e) and 2D Raman mapping at 677 and 789 cm^–1^ for TZL5 (b, c) and TZL9 (f, g) glasses after fs-laser irradiation. Dotted lines indicate intensity variations, correlating with waveguide structural changes. (d, h) Mapping of the *I*(TeO_3_)/*I*(TeO_4_) ratio retrieved by integrating the normalized spectral region. (i) Model of transverse refractive index profiles for TZL5 and TZL9 samples, highlighting differences in mode confinement.

Figure 7b,c,f,g shows the 2D Raman mapping of the regions around 677 cm^–1^ (TeO_4_) and 789 cm^–1^ (TeO_3_), highlighting the fs-laser-induced structural changes in the glass structure. Brighter/darker regions indicate stress-affected areas, while darker/brighter regions correspond to unaffected glass, consistent with the waveguide profile. The analysis focuses on the intensity and central wavenumber variations across the waveguide cross section, particularly in the TeO_4_ and TeO_3_ bands, which reflect changes associated with structural modifications and the refractive index profile [REF]. This is further supported by the net volume expansion indicated by the compressive stress field and the observed variation in intensity in bands at 677 and 789 cm^–1^, which can be associated with the breaking of Te–O–Te bonds or structural transformation (TeO_3_ + NBO → TeO_4_) in both the head and tail of the tear-shaped laser tracks, confirming modifications within the 3D glass network. Additionally, differences in the focal volume and surrounding eye-shaped contours between structures written with different La^3+^ concentrations (TZL5 and TZL9) suggest variations in structural configuration, likely corresponding to areas of density contrast and refractive index modulation.

This phenomenon originates from two main factors: first, in the laser’s focal volume, the formation of TeO_3_ or TeO_4_ associated with oxygen vacancies and O_2_ generation that creates mobile oxygen atoms. These atoms diffuse from regions with a higher oxygen bonding concentration to those with a lower oxygen presence, resulting in oxygen-deficient zones within the modified structure. Second, ion migration leads to an increase in the high-frequency band intensity corresponding to NBO formation. Consequently, the glass expands across the tear-shaped structure, with the Raman map suggesting Te–O–Te bond breaking, structural transformation (TeO_3_ + NBO → TeO_4_), or Zn^2+^ migration toward the tail of the modified region, as already proposed.^26,27^

This behavior is consistent with the Raman shifting to higher vibrational energies under such stress (see Figure 7a,b). The stress distribution observed in the Raman mapping mirrors the SEM images, where TZL5 exhibited a more confined cross section, while TZL9 showed a broader modification area. Dotted white lines in Figure 7b,c (for TZL5) and Figure 7f,g (for TZL9) illustrate how the intensity of these bands aligns with the waveguide structural profile, suggesting consistency in the structural changes induced by the laser across different glass regions.

Figure 7d,h shows normalized Raman spectra of the intensity ratio (*I*(TeO_3_)/*I*(TeO_4_)) for the waveguide writing configuration. The Raman mapping further reveals that the intensity ratio (*I*(TeO_3_)/*I*(TeO_4_)) reached a maximum of ∼1.23 for TZL5, while it decreased to ∼1.11 for TZL9. This change in the *I*(TeO_3_)/*I*(TeO_4_) ratio in the waveguide depends on the initial relative concentrations of TeO_3_ and TeO_4_ in each TZL glass, as illustrated in Figure 7b,c,f,g. These results further support the interpretation that the structural transformation from TeO_3_ + NBO to TeO_4_ is driven by the shock pressures generated during fs-laser exposure, leading to refractive index changes and distinct waveguide profiles in TZL5 and TZL9 glasses.

Figure 8a illustrates the experimental setup for waveguide fabrication, and Figure 8b shows the standard end-face coupling setup used to assess the near-field mode intensity. Figure 8c,d shows the near-field mode profiles at 633 nm for TZL5 and TZL9 samples across various energy levels (207–271 nJ), revealing the optical performance of the waveguides.

**Figure 8:**
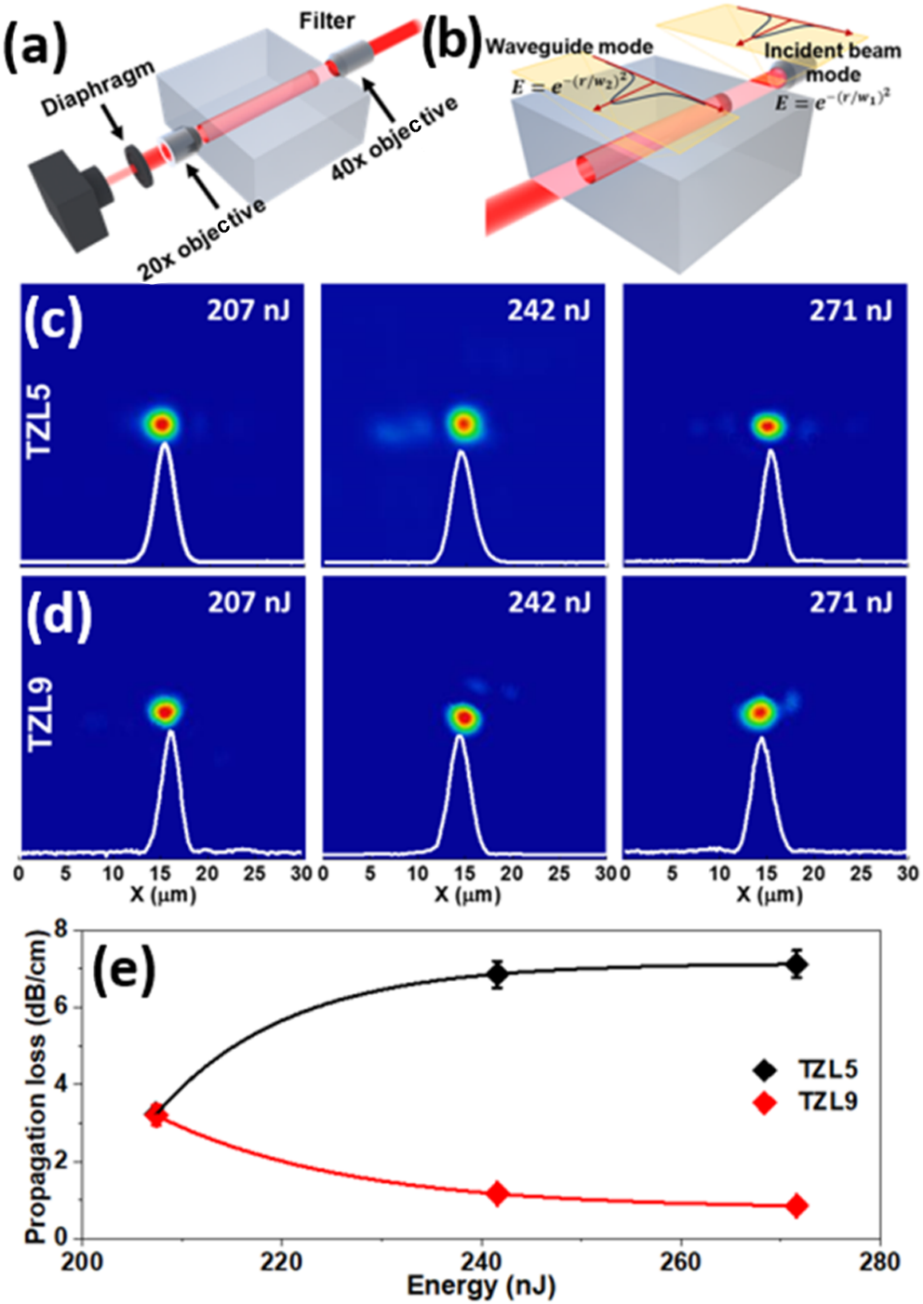
(a) Schematic of the experimental setup for waveguide fabrication: continuous waveguides are created by tightly focusing an ultrafast laser beam within the TZL glass. (b) Waveguide formation occurs by applying multiple laser pulses at relatively high speeds. (c, d) Experimental near-field mode profiles of the light guide at 633 nm emerging from each waveguide for different energies. (e) Propagation loss as a function of energy for TZL5 (black markers) and TZL9 (red markers) waveguides.

Due to the enhanced confinement provided by the damage lines, the TZL waveguides exhibit low propagation losses across the pulse energy range of 207–271 nJ. However, TZL5 and TZL9 demonstrate opposite behaviors, attributed to their structural differences. Propagation losses, encompassing both guiding and Fresnel losses, were measured using the overlap integral method:

3 $$\eta \;\left(dB\right)=-10\log_{10}\left[\frac{{\left(\int E_{g}E_{f}^{*}dxdy\right)}^{2}}{\int E_{g}E_{g}^{*}dxdy\int E_{f}E_{f}^{*}dxdy}\right]$$

where *E*_g_ represents the field profile of the mode in the waveguide and *E*_f_ is the field profile of the mode in the laser. This method accounts for the overlap between the guided mode and the input laser mode, focusing on the coupling efficiency.

For TZL5, propagation losses increased from 3.22 to 7.12 dB/cm as the energy rose, while in TZL9, losses decreased from 3.20 to 0.85 dB/cm (Figure 8e). This contrasting behavior can be attributed to the *I*(TeO_3_)/*I*(TeO_4_) ratio, which reflects the stability and flexibility of each glass network, as shown in the Raman analysis. A higher *I*(TeO_3_)/*I*(TeO_4_) ratio reflects a predominance of TeO_3_ units and NBOs, resulting in regions that are more polarizable but are structurally less stable. In TZL5, these less stable regions become more susceptible to structural rearrangement as laser energy increases, leading to a low degree of local densification. Consequently, an increase in scattering sites reduces light confinement, resulting in narrower mode profiles and higher propagation losses. Additionally, the lower TeO_3_ content (prior to waveguide fabrication) in TZL5 limits the effectiveness of the TeO_3_ + NBO to TeO_4_ conversion driven by laser-induced shock pressures and temperature gradients, further contributing to its structural instability and increased propagation losses. In contrast, TZL9, with a lower *I*(TeO_3_)/*I*(TeO_4_) ratio, has a higher concentration of TeO_4_ units, creating a more rigid and stable structure that resists changes under both high laser energy and thermal effects. This stability supports a broader mode profile, enhancing light guidance and reducing propagation losses. At the same time, the greater presence of TeO_3_ (prior to waveguide fabrication) in TZL9 facilitates the TeO_3_ to TeO_4_ conversion under these conditions, promoting greater structural resilience and maintaining low optical losses at higher pulse energies.

Overall, the *I*(TeO_3_)/*I*(TeO_4_) ratio plays a critical role in waveguide performance, with Raman spectroscopy highlighting how TeO_3_ and TeO_4_ structures impact optical losses. The La^3+^-modulated glass composition, as seen in Raman spectra, is essential for optimizing stability and confinement, with a higher TeO_4_ concentration in TZL9 improving light guidance and reducing losses, whereas the TeO_3_-rich structure in TZL5 tends toward increased losses.

The propagation losses obtained in TZL9, which decrease to as low as 0.85 dB/cm at higher pulse energies, are comparable to or even lower than those reported for femtosecond laser-written waveguides in other tellurite-based glasses. In previous studies involving TeO_2_–ZnO glasses doped with rare-earth ions, losses typically ranged from 1.0 to 2.0 dB/cm under similar laser inscription conditions.^45^ Additional reports on waveguides in germanate and tellurite glasses confirm the viability of fs-laser micromachining in such matrices, supporting the relevance of our findings.^46^ These comparisons emphasize the favorable performance of La-rich TZL9 glasses, particularly when processed at high-repetition rates and moderate pulse energies, and reinforce the role of the La_2_O_3_ content and the *I*(TeO_3_)/*I*(TeO_4_) ratio in achieving low-loss waveguides.

A 2D Gaussian permittivity distribution was employed to model the spatial modifications in the glass to simulate the influence of laser processing on waveguide formation. Figure 9a–d shows the simulated cross-sectional electric field intensity distributions for the TE mode in TZL5 (a, b) and TZL9 (c, d) glasses at low and high laser energies. By comparison of these simulations with the experimentally observed modes (Figure 8), it is clear that the fabrication energy strongly influences the refractive index distribution.

**Figure 9:**
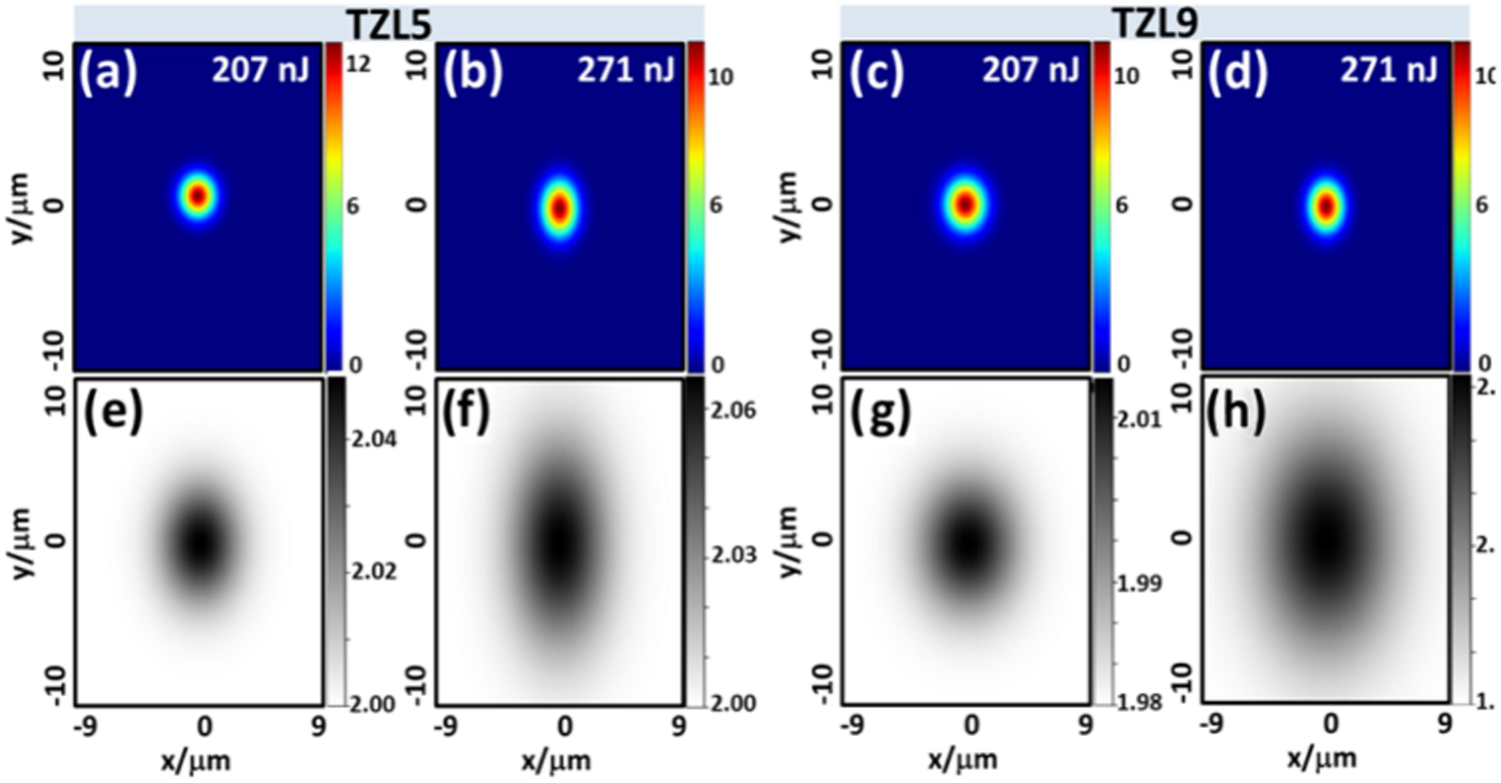
Cross-sectional electric field intensity distribution of the TE-supported mode in the microfabricated waveguide for TZL5 glass at (a) 207 nJ and (b) 271 nJ and for TZL9 glass at (c) 207 nJ and (d) 271 nJ. Corresponding simulated refractive index profiles using a 2D Gaussian model for TZL5 glass at (e) 207 nJ and (f) 271 nJ and for TZL9 glass at (g) 207 nJ and (h) 271 nJ.

To account for different energy conditions, the standard deviations of the Gaussian profile were derived from the micrographs in Figure 5, and the peak permittivity values were refined to match the experimental mode profiles closely. The resulting simulated index maps, shown in Figure 9e–h, reveal a positive index change following a Gaussian-like shape. The simulations suggest that the refractive index in TZL5 glass remains relatively unchanged between low and high energies, whereas TZL9 glass exhibits a more pronounced index increase at higher energies. This enhanced response in TZL9 correlates with its lower propagation losses, consistent with previous observations, where the densification network structure plays a key role in improving waveguide performance.

Moreover, these findings corroborate our earlier hypothesis that the ratio *I*(TeO_3_)/*I*(TeO_4_) plays a critical role in establishing the baseline refractive index and dictating its subsequent laser-induced modifications. While TZL5 glass, with a relatively higher ratio, shows minimal index changes under varying energies, TZL9 glass, with a lower ratio, exhibits more pronounced densification and a higher refractive index contrast, thereby explaining its lower propagation losses and overall enhanced waveguide performance.

## 4 Conclusions

This study demonstrates that La^3+^ doping and the *I*(TeO_3_)/*I*(TeO_4_) ratio significantly influence the structural and optical properties of TeO_2_–ZnO–La_2_O_3_ (TZL) glass waveguides fabricated by femtosecond laser microfabrication. Raman analysis showed that La^3+^ enhances network stability by promoting TeO_4_ formation through a structural transformation of TeO_3_ + NBO → TeO_4_, driven by high-pressure shock waves and thermal effects from the laser. This transformation reduces nonbridging oxygens (NBOs), with TZL9 displaying a lower *I*(TeO_3_)/*I*(TeO_4_) ratio (∼1.11) compared to TZL5 (∼1.23). This stability led TZL9 waveguides to exhibit broader mode profiles, lower optical scattering, and reduced propagation losses, decreasing from 3.20 to 0.85 dB/cm as pulse energy increased from 207.4 to 271.6 nJ. Conversely, the TeO_3_-rich structure in TZL5, more susceptible to laser-induced rearrangements, resulted in narrower profiles and increasing losses from 3.22 to 7.12 dB/cm over the same energy range. Complementarily, simulations further confirmed our experimental findings. The modeled electric field and refractive index profiles indicate that enhanced densification in TZL9 at higher pulse energies improves light confinement. These findings advance the current state of the art by establishing a direct correlation between spectroscopic structural transformations and the optical waveguide performance in La^3+^-doped tellurite glasses. The integration of Raman mapping, loss analysis, and numerical modeling offers a powerful framework for optimizing fs-laser-written photonic devices. In summary, this work provides both fundamental insight and practical strategies for engineering low-loss, compositionally tunable waveguides in nonlinear optical glasses.
